# Identification of Promoter Region Markers Associated With Altered Expression of Resistance-Nodulation-Division Antibiotic Efflux Pumps in *Acinetobacter baumannii*

**DOI:** 10.3389/fmicb.2022.869208

**Published:** 2022-05-19

**Authors:** Mireia López-Siles, Michael J. McConnell, Antonio J. Martín-Galiano

**Affiliations:** Intrahospital Infections Laboratory, National Center for Microbiology, Instituto de Salud Carlos III (ISCIII), Madrid, Spain

**Keywords:** resistome, promoter, repressor, insertion sequence, systems biology, nosocomial infection, operator

## Abstract

Genetic alterations leading to the constitutive upregulation of specific efflux pumps contribute to antibacterial resistance in multidrug resistant bacteria. The identification of such resistance markers remains one of the most challenging tasks of genome-level resistance predictors. In this study, 487 non-redundant genetic events were identified in upstream zones of three operons coding for resistance-nodulation-division (RND) efflux pumps of 4,130 *Acinetobacter baumannii* isolates. These events included insertion sequences, small indels, and single nucleotide polymorphisms. In some cases, alterations explicitly modified the expression motifs described for these operons, such as the promoter boxes, operators, and Shine-Dalgarno sequences. In addition, changes in DNA curvature and mRNA secondary structures, which are structural elements that regulate expression, were also calculated. According to their influence on RND upregulation, the catalog of upstream modifications were associated with “experimentally verified,” “presumed,” and “probably irrelevant” degrees of certainty. For experimental verification, DNA of upstream sequences independently carrying selected markers, three for each RND operon, were fused to a luciferase reporter plasmid system. Five out of the nine selected markers tested showed significant increases in expression with respect to the wild-type sequence control. In particular, a 25-fold expression increase was observed with the IS*Aba*1 insertion sequence upstream the *adeABC* pump. Next, overexpression of each of the three multi-specific RND pumps was linked to their respective antibacterial substrates by a deep *A. baumannii* literature screen. Consequently, a data flow framework was then developed to link genomic upregulatory RND determinants to potential antibiotic resistance. Assignment of potential increases in minimal inhibitory concentrations at the “experimentally verified” level was permitted for 42 isolates to 7–8 unrelated antibacterial agents including tigecycline, which is overlooked by conventional resistome predictors. Thus, our protocol may represent a time-saving filter step prior to laborious confirmation experiments for efflux-driven resistance. Altogether, a computational-experimental pipeline containing all components required for identifying the upstream regulatory resistome is proposed. This schema may provide the foundational stone for the elaboration of tools approaching antibiotic efflux that complement routine resistome predictors for preventing antimicrobial therapy failure against difficult-to-threat bacteria.

## Introduction

Prompt and precise identification of genetic determinants that contribute to antibiotic resistance, the resistome, in difficult-to-treat bacteria can facilitate the administration of the most effective therapy. Cost decreases in DNA sequencing have further promoted the development of several computational protocols that predict antibiotic resistance at a whole-genome level (Gupta et al., 2014; de Man and Limbago, 2016; Alcock et al., 2020; Bortolaia et al., 2020) with a reported accuracy comparable to antibiograms determined using traditional microbiological techniques. Resistome identification often involves at least three elements: (a) a database of resistance determinants, either whole genes or specific mutations; (b) an algorithm that accurately detects the determinants in the genome sequence of interest; and (c) a controlled language that links genetic determinants of resistance to specific antibiotics (Alcock et al., 2020).

Isolates with identical predicted resistomes can, however, demonstrate different antibiograms and/or responses to antimicrobial therapy (Gerson et al., 2018). False predictions due to determinants that escape current algorithms can cause therapeutic failure, leading to increases in treatment cost and adverse outcomes. The identification of genetic markers underlying the constitutive upregulation of efflux pumps is considered the most significant challenge for future resistome predictors (Jeukens et al., 2017; Boolchandani et al., 2019; Mahfouz et al., 2020). Overexpression of otherwise repressed efflux pumps can reduce the cytoplasmic concentration of an antibiotic to ineffective levels (Kapp et al., 2018). Pump upregulation can be achieved by alterations in either repressor proteins (Gerson et al., 2018) or in upstream sequences of pump genes involved in gene expression (Olliver et al., 2005; Baylay and Piddock, 2015). Therefore, prediction of efflux-based resistance only by gene presence can lead to inaccurate interpretations. The multiplicity of DNA elements affecting gene transcription and translation makes the automated screening of upstream sequences for resistance traits a formidable task.

Efflux pump upregulation is a prominent resistance mechanism in *Acinetobacter baumannii* (Vila and Pachon, 2011; Cag et al., 2016), a nosocomial pathogen of high priority for international health organizations (Tacconelli et al., 2018; Rello et al., 2019). The resistance-nodulation-division (RND) system is the most relevant and extensively studied efflux pump family in this species (Lin et al., 2015). RND complexes demonstrate multi-specificity, and consequently single genetic events that produce their upregulation can increase resistance to several unrelated antibiotics (Nikaido and Pagès, 2012). Nearly, all *A. baumannii* isolates harbor three RND types encoded by the *adeABC* (Magnet et al., 2001), *adeFGH* (Coyne et al., 2010), and *adeIJK* (Damier-Piolle et al., 2008) operons. Their expression is tightly controlled by cognate regulatory repressors, namely AdeRS (Marchand et al., 2004; Chang et al., 2016), AdeL (Coyne et al., 2010), and AdeN (Rosenfeld et al., 2012), respectively, that bind to DNA operator motifs upstream of the operon. Regular and active repression of RND operon transcription prevents diminishment of bacterial fitness since high pump levels may lead to increased metabolic requirements, proton motive force exhaustion, and imbalances in the sessile-to-planktonic equilibrium (Leus et al., 2018). Nevertheless, genetic changes leading to dysregulation of this control can still be advantageous under the antibiotic- and disinfectant-rich environment of healthcare centers (Higgins et al., 2010; Machado et al., 2018). Mutations affecting full translation or DNA binding in repressor proteins of RND pump operons have been associated with MIC increases to several antibiotics (Gerson et al., 2018). Moreover, alterations have also been associated with resistance in upstream untranslated regions of *adeIJK* (Zang et al., 2021).

The substantial body of knowledge gained for *A. baumannii* RND regulation has not been transferred to automated resistome tools. The exclusion of untranslated upstream factors producing constitutive RND expression can lead to inappropriate therapy, in particular for some last-resort therapies, such as tigecycline. In this study, we provide several proofs of concept required for overcoming limiting steps prior to integrating resistome tools based on upstream and coding sequences.

## Materials and Methods

### Sequence Identification and Management

The first gene of the three RND pump operons was screened by nBLAST, with ≥80% identity and ≥95% alignment length thresholds, in non-anomalous *A. baumannii* genomes stored in the Assembly database (Kitts et al., 2016). E5A70_10260 (*adeA*), A1S_2304 (*adeF*), and A1S_2735 (*adeI*) ORFs from *A. baumannii* ATCC17978 were used as reference query sequences for the nBLAST search. Then, 500 nt upstream sequences were extracted for each gene and isolate, if not discontinued by contig-limits, and subjected to clustering by CD-HIT (Fu et al., 2012) on the stringent 100% identity and 100% alignment length basis to detect allelicity. All alleles were aligned to their respective wild type (WT) sequences with Muscle v3.8.31 (Edgar, 2004). Insertion sequences (ISs) were detected with ISFinder (Siguier et al., 2012). For alleles alignable with the whole WT sequence but showing >20 SNPs to the WT sequence, the species carrying the most significant hit was searched by nBLAST against the whole *Acinetobacter* genus in the NCBI nucleotide collection (nr/nt) database. If not explicitly reported in the literature, the most probable −35 and −10 promoter box sequences were predicted by Pattern locator (Mrazek and Xie, 2006) applying the {TTGACA}[2](N)[15–20]{TATAAT}[2] motif. Shine-Dalgarno sequences were those located between −15 and −3 positions with respect to the start codon that showed the lowest free energy of the pairing with respect to the consensus anti-ribosome binding site sequence (CCTCCT) using the RNAcofold algorithm, available in the Vienna RNA 2.0 suite (Lorenz et al., 2011). If more than one candidate Shine-Dalgarno sequence was identified, the one closest to the optimal 7 nt spacer to the start codon was selected. DNA bending was calculated using Bend-it (Vlahovicek et al., 2003), applying a curvature window size of 31 nt. The minimum free energy (MFE) of RNA secondary structures of both whole alleles and WT sequences containing SNPs in isolation was calculated by the RNA-fold program of the Vienna RNA 2.0 suite (Lorenz et al., 2011). For MFE calculation, only the sequence section from the experimental (*adeABC*, −403) (Kröger et al., 2018) or theoretical (*adeFGH*, −188; *adeIJK*, −31) start transcription site to the −1 position, i.e., the transcribed zone, was considered.

Sequence types (STs) were assigned using the Oxford scheme (Bartual et al., 2005) by identification of perfect matches (100% identity over 100% aligned length) by nBLAST using allelic information from the official MultiLocus Sequence Typing (MLST) site.^1^ Spatial and time isolate metadata were collected from the Biosample database (Barrett et al., 2012). Average nucleotide identity (ANI) at genome level was calculated with OrthoANI (Yoon et al., 2017).

### Construction of Chimeras and Experimental Activity Assessment

Selected upstream sequences were synthetized *ab initio* by Thermo Fisher Scientific Inc. (Massachusetts, United States) flanked by *BamHI* and *NotI* target sequences. Synthesized DNA fragments and the pLPV1Z plasmid (Lucidi et al., 2019) were cleaved with appropriate restriction enzymes and, after ligation, electroporated into *Escherichia coli* DH5α. Constructions were verified by Sanger sequencing and then introduced into *A. baumannii* ATCC 17978 by electroporation as previously reported (Lucidi et al., 2019). To test the promoter activity of individual alleles, *A. baumannii* cells were grown overnight at 37°C in LB medium with gentamycin, then cultures were diluted 1:100, and incubated under the same conditions but without gentamycin for 6 h. The OD_620_ and luminescence were measured at this point. Relative luminescence units (RLUs) for each sample were normalized to OD_620_, the background (culture with no plasmid) subtracted, and then divided by the same value obtained for the intra-experiment WT control.

### Conventional Resistome Prediction

The conventional resistome, involving coding sequences, was determined by CARD2020 (Alcock et al., 2020). Only “perfect” and “strict” hits were considered. Sequence quality “high quality/coverage” was applied. Nudge loose hits to strict were excluded.

## Results

### Analysis of the Allelic Variability of RND Upstream Sequences

The sequence variability of upstream regions of operons coding the three principal RND pumps of *A. baumannii* (AdeABC, AdeFGH, and AdeIJK) was screened. For that, full sections of 500 bp upstream of these operons were identified for 89%–99% of *A. baumannii* genomes in a sample of 4,130 isolates. These isolates represented 352 STs previously reported by PubMLST (Jolley et al., 2018). Identical upstream sequences were unified into “upstream alleles.” For the three operons considered, there was a dominant upstream allele (covering 52%–64% of the total of isolates) that involved a large number of STs and was therefore considered the WT sequence. There was large disparity for a number of upstream alleles and their average genetic distance to the WT between the three operons (*adeABC* > *adeFGH* > *adeIJK*; Table 1). This suggests the existence of different intensities for selective pressure acting on the regulation of *A. baumannii* RND pumps.

**Table 1:** Resistance-nodulation-division (RND) gene coverage in sequenced genomes and allelicity data.

Property	*adeABC*	*adeFGH*	*adeIJK*
% isolates in which the first operon gene were detected	92.1	99.2	99.3
% isolates in which 500 nt upstream the first operon gene were available	89.3	98.6	98.2
Number of upstream alleles	213	110	80
Average (±SD) allelic SNPs	9.3 ± 8.2	7.1 ± 9.7	4.6 ± 7.4
% isolates showing the WT allele	62.4	52.3	62.6
Number of identified tags	203	172	87
Number of alleles (isolates) with ISs	17 (40)	1 (1)	7 (7)
% alleles with a bending peak ≥2°/turn with respect to WT^a^	61	18	88
MFE mRNA secondary structure of WT (Kcal/mol)	−61.0	−41.2	−3.6
% alleles with mRNA mfe ≥ 5% with respect to WT^b^	71.6	13.9	6.9

a Only alleles showing no indels respect to the WT sequence. Maximal bending differences between equivalent positions respect to WT were considered.

b Only alleles showing no indels respect to the WT sequence in the transcribed section were considered.

**Figure 1:**
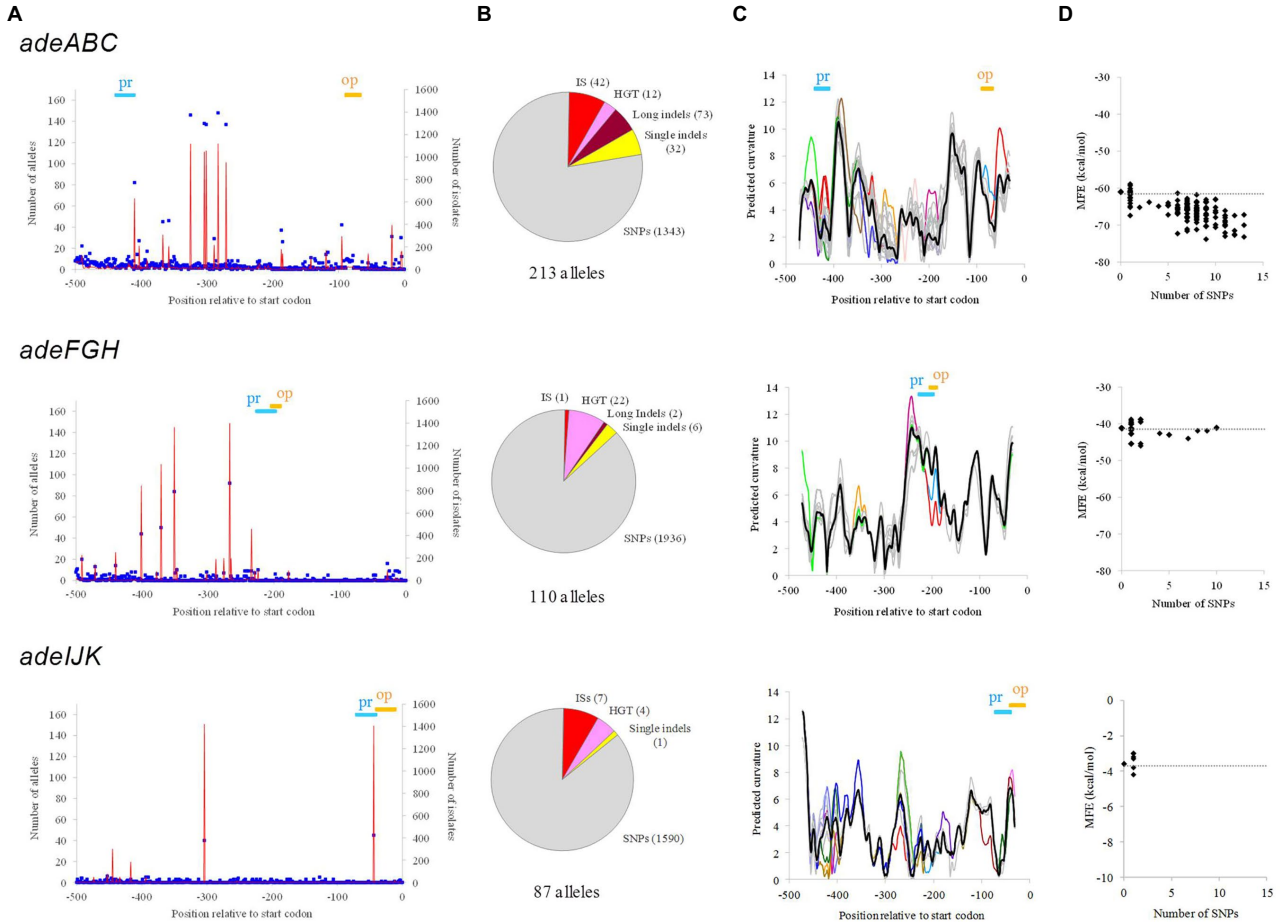
Genetic and structural properties of upstream alleles of *Acinetobacter baumannii* RND-encoding operons. **(A)** Location of RND upstream positions showing SNPs. Polymorphisms due to ISs were not included. Allelic occurrence (blue points) and the number of isolates involved (red line) are shown. The positioning of promoter (Pr) and operator (Op) motifs is indicated. **(B)** Number of alleles and raw genetic events observed for each operon. If more than one type of genetic event pertained the same position, only the most relevant was considered applying the following prevalence: ISs > horizontal gene transfer (HGT) > long indels > single indels > SNPs. **(C)** DNA bending calculations for all allelic sequences showing no indels respect to the WT. Curvature is expressed in degrees per helical turn. Thick black line: allelic average (nearly identical to the WT values); superimposed alleles are in grey except for those showing maximal divergences of ≥2 degrees/turn with respect to the allelic average or ≥1 degree/turn in key motifs, which were color-highlighted. The positioning of promoter (Pr) and operator (Op) motifs is also indicated. **(D)** Minimum free energy (MFE) of the mRNA structure for all allelic sequences showing no indels respect to the WT. Black dots indicate MFE values and the number of SNPs respect to the WT for each allele. The MFE value for the WT is indicated (grey line).

Within alleles, a total of 487 non-redundant genetic alterations (termed here determinants, markers, or tags) were identified with respect to the reference WT sequence in the three datasets (Supplementary Table S1). Up to 54% of the isolates evaluated carried at least one determinant for one of the three RND pumps. Determinants showed a wide value range for parameters, such as isolate occurrence, predictable degree of severity, distance to start codon, and type of genetic event (Figures 1A,B).

Of note, 19 alleles involving 30 isolates and six official STs showed >20 SNPs. These alleles were more similar to sequences from *Acinetobacter nosocomialis* or *Acinetobacter pittii* (Supplementary Table S2), two species considered less pathogenic than *A. baumannii*. Most of these alleles corresponded to original species misassignation. Only one allele involving four isolates appeared to be from an *A. baumannii* isolate according to whole genome ANI analysis. These four isolates were collected in Thailand in 2018 and were nearly identical (all-against-all ANI ≥ 99.8%), which indicates outbreak or intra-patient microevolution sampling. These isolates were closer to the *A. baumannii* reference sequence (ab736 strain, ANI = 97.6%, where ANI > 95% is accepted as same species) than to *A. nosocomialis* and *A. pittii* isolates (ANI < 92%). In these isolates, two similarity swaps between *A. nosocomialis* and *A. baumannii* sequences, i.e., 5′ and 3′ potential recombination points were detected. These located at 502-GGCGTTTTTAAAC-514 of the *adeS* ORF and the 941-GAGCC-945 nucleotides of the *adeA* ORF (Supplementary Figure S1A). Thus, the genetic exchange affected both translated and untranslated elements. On the one hand, the resulting hybrid AdeS, AdeR, and AdeA polypeptides shared 98%–99% similarity with the *A. baumannii* reference homolog. Whether these scarce residue changes suffice to affect protein activity of the mosaic protein should be assessed. On the other hand, in contrast, the impact on expression in the recombinants appears more explicit since *A. nosocomialis* genuine mutations affected key motifs and nucleic acid structure of the *adeABC* upstream sequence (Supplementary Figures S1B,C). This suggests different AdeABC regulation in the inter-species recombinant *A. baumannii* isolates, which may have clinical consequences.

To assess the potential effect of the identified tags on pump overexpression, their context with respect to 16 key motifs explicitly associated with pump expression was evaluated. These motifs included promoter boxes, repressor operators, and Shine-Dalgarno sequences (Table 2). Interestingly, at least for those markers affecting key motifs, genetic tags involved a fraction of isolates from different STs and unrelated geotemporal sampling data. The global absence of a clear clonal origin for these determinants suggests convergent evolution and/or horizontal transfer rather than pure vertical inheritance. However, it is unknown whether maintenance of tags that involve constitutive RND expression is favored by some specific genetic backgrounds.

**Table 2:** Upstream motifs associated to RND pump expression.

Operon	Motif	Start	End	Length	WT sequence	Identification method^a^	Reference
*adeABC*	Promoter-35 box	−438	−433	6	TTATCA	Primer extension	Marchand et al., 2004
	Promoter-10 box	−415	−411	5	CGTCA	Primer extension	Marchand et al., 2004
	Start transcription site	−403	−403	1	C	Primer extension	Marchand et al., 2004
	AdeR binding operator repeat 1	−88	−79	10	CTCCACACTT	EMSA	Chang et al., 2016
	AdeR binding operator repeat 2	−77	−68	10	CTCCACACTT	EMSA	Chang et al., 2016
	Shine-Dalgarno region	−8	−3	5	TGGACA	RNAcofold	This work
*adeFGH*	Promoter-35 box	−226	−221	6	TTGTTA	Bprom	Coyne et al., 2010
	Promoter-10 box	−205	−198	8	TGTTATCA	Bprom	Coyne et al., 2010
	AdeL binding operator	−203	−191	13	TTATCAAATTTAA	Presence of LTTR box	Coyne et al., 2010
	Shine-Dalgarno region	−13	−8	6	CGGTGG	RNAcofold	This work
*adeIJK*	Promoter-35 box	−70	−65	6	ATTACA	TSSs, PatLoc	This work, Kröger et al., 2018
	Promoter-10 box	−46	−41	6	TAAAAA	TSSs, PatLoc	This work, Kröger et al., 2018
	AdeN binding operator	−39	−12	28	CAAATATATTTTTAGATTTTATCTAAAC	Manual inspection	Rosenfeld et al., 2012
	Shine-Dalgarno region	−13	−8	6	ACGAGG	RNAcofold	This work

a EMSA, electrophoretic mobility shift assay.

Some upstream alleles showed abrupt mismatching to the WT, which were due to the presence of ISs. These events differed with respect to (i) the distance between the IS insertion site and the start codon, where ISs can cause complete removal of central elements such as promoters and/or operators; and (ii) the IS family involved, either IS*Aba*1 or IS*Aba*4. Both IS family sequences carry strong promoters: TTAGAA-N_16_-TTATTT and TAACTA-N_17_-TTTCTT, respectively.

DNA bending and mRNA secondary structure of alleles were also analyzed. DNA bending can alter expression (Agustiandari et al., 2011) by modifying DNA accessibility and/or recognition by the RNA polymerase and repressors. A substantial fraction of upstream alleles that could be aligned to the WT allele over the full length (<20 SNPs, no indels) showed maximal bending differences over 2° per turn (Table 1; Figure 1C). In some cases, these high curvature difference peaks affected the promoter and operator zones. The stability of the mRNA secondary structure can also modulate expression (Del Campo et al., 2015) by changing transcription rate, translation efficiency, and hydrolysis by RNases. WT alleles from the three genes showed distant predicted values for maximum free energy at the mRNA level that in some alleles was altered by more than 10% (Table 1; Figure 1D).

### Experimental Verification of DNA Marker-to-Phenotype Relationships

**Figure 2:**
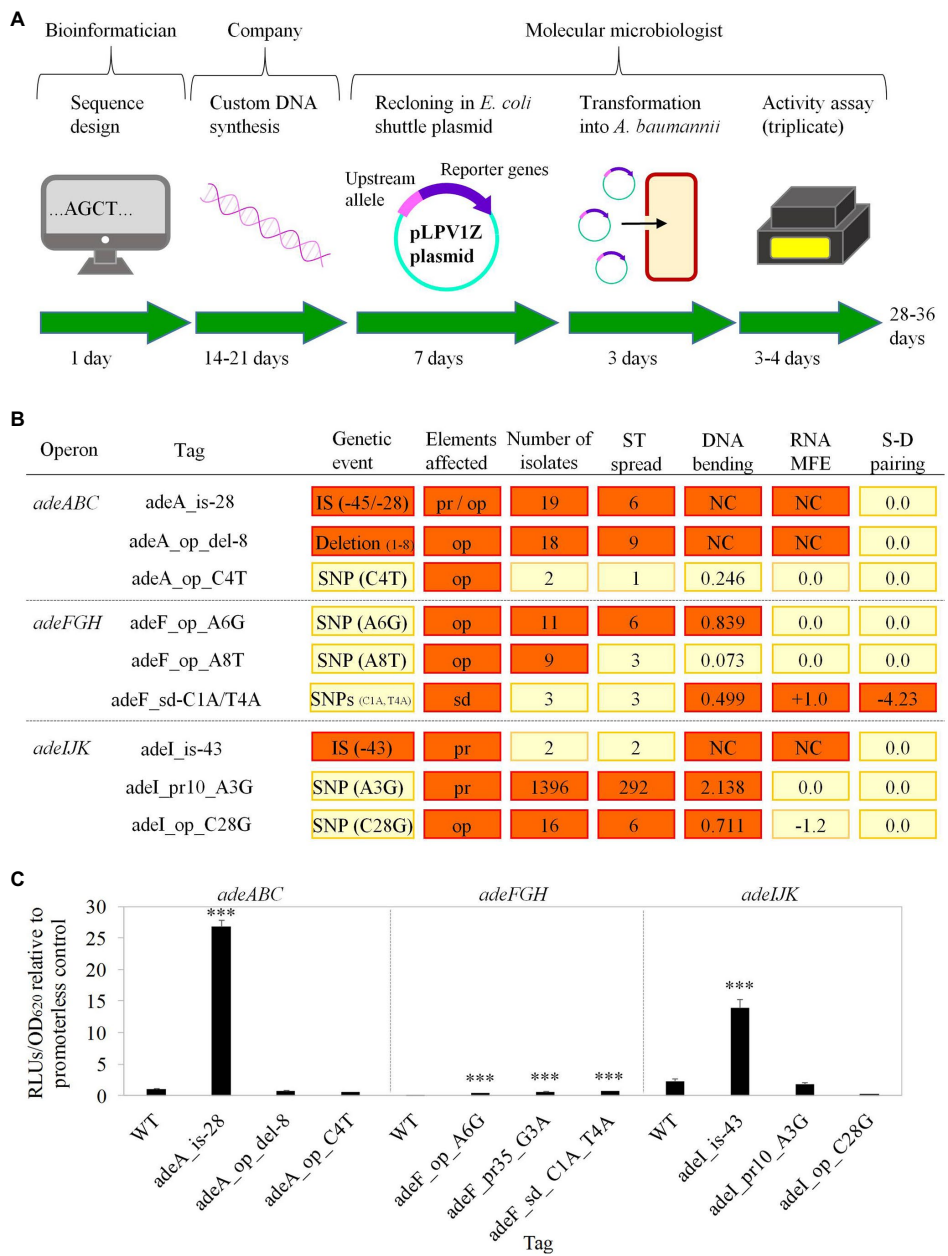
Experimental validation of selected tags. **(A)** Timeline schema of the experimental validation pipeline. Stages, human staff involved, and their estimated time duration are shown. **(B)** Tags selected for experimental evaluation. Tag nomenclature include the first gene of the pump operon followed by either the label “-is” plus the insertion distance to start codon (for ISs), or the element affected (op: operator; pr: promoter; and sd: Shine-Dalgarno sequence) plus the “del” label plus the length of the removed section (for deletions) or the nucleotide change (for point mutations). For clarity, values for parameter criteria utilized for tag prioritization are highlighted in red when deemed relevant: deletions or IS (genetic event); opt, pr, and sd (elements affected); ≥5 isolates carrying the tag (number of isolates); ≥5 different STs with isolates carrying the tag (ST spread); ≥0.5 degrees per turn difference respect to the WT (DNA bending); ≥1 Kcal/mol differences in RNA MFE (RNA MFE); and ≥1 Kcal/mol differences in Shine-Dalgarno (S-D) and anti-S-D ribosomal sequence pairing (S-D pairing). IS alleles were clustered if the insertion point was so proximal that the same motifs were affected. The number of isolates that harbor the tag is shown together with the number of alleles involved under brackets. Isolates not included in formal STs were considered together as a single unit for the ST count estimation. DNA bending, RNA MFE, and S-D pairing columns indicate maximal differences of the tag-carrying sequence respect to the WT in degrees per turn for the former, and Kcal/mol for the rest parameters. NC, non-comparable. **(C)** Expression activity of prioritized alleles measured by luminometry. Fold-changes of normalized RLUs associated to the alleles respect to the promoterless cells are shown. Cells containing plasmids with WT upstream sequences for *adeABC*, *adeFGH*, and *adeIJK* pumps showed 103 ± 9%, 5 ± 2%, and 220 ± 46% expression values, with respect to the original promoterless plasmid, respectively. Data are the averages ± SDs of four independent experiments. Statistical significance was calculated using the two-tailed Student’s *t*-test. Significant differences are indicated: ^***^p < 0.001.

Based on findings from previous studies, the effect of some of the markers above on pump expression, and likely on resistance, can be theoretically presumed. However, certainty on upregulation caused by these markers can only be obtained by experimental corroboration under an isogenic background. Given the high technical difficulty in introducing chromosomal modifications in *A. baumannii*, a plasmid mid-throughput screening was developed with the aim of validating markers that result in gene upregulation. An illustrative schema for such screening that contains the procedural steps, expertise required, and expected timescale is provided in Figure 2A. Our method involved the fusion between synthesized DNA carrying the marker to a luciferase report system.

This plasmid reporter system has been previously validated for characterizing gene expression dynamics under multiple experimental conditions (Lucidi et al., 2019) and used to assess expression changes in RND pumps from *A. baumannii* reference strains (Prieto Martin Gil et al., 2021). In our hands, the series of steps from DNA design to measurement of expression activity can be accomplished with an average cost of 150€ per marker in a turnaround time of 28–36 working days.

Since the experimental evaluation of the whole determinant catalog is not feasible, the protocol was evaluated using three selected markers per operon. The selected genetic tags were prioritized in order to optimize coverage of different ranges of the nature of the genetic changes, type of DNA motif affected, DNA curvature and mRNA structural alterations, isolate occurrence, and clonal distribution (Figure 2B).

Significant differences of up to 25-fold between the expression of five genetic tags, out of nine, and both promoterless plasmid cells and their respective WT controls were observed (Figure 2C). The most active markers for *adeABC* and *adeIJK* corresponded to IS insertions. Notably, the results obtained with ISs allowed us to verify our plasmid-luciferase experimental model, after observations of similar genomic arrangements involving upregulation of other resistance determinants such as carbapenemases (Corvec et al., 2003, 2007; Turton et al., 2006; Adams et al., 2010). Besides, selected mutations in the repressor-binding operator and the Shine-Dalgarno sequence of *adeFGH* produced notable increments in the expression of the reporter gene downstream. Altogether, 42 isolates in the dataset harbor at least one of these experimentally validated upregulating determinants (Supplementary Table S3).

### Analysis of RND Regulatory Resistance in Testing Isolates: Algorithm Flowchart and Ontology

**Figure 3:**
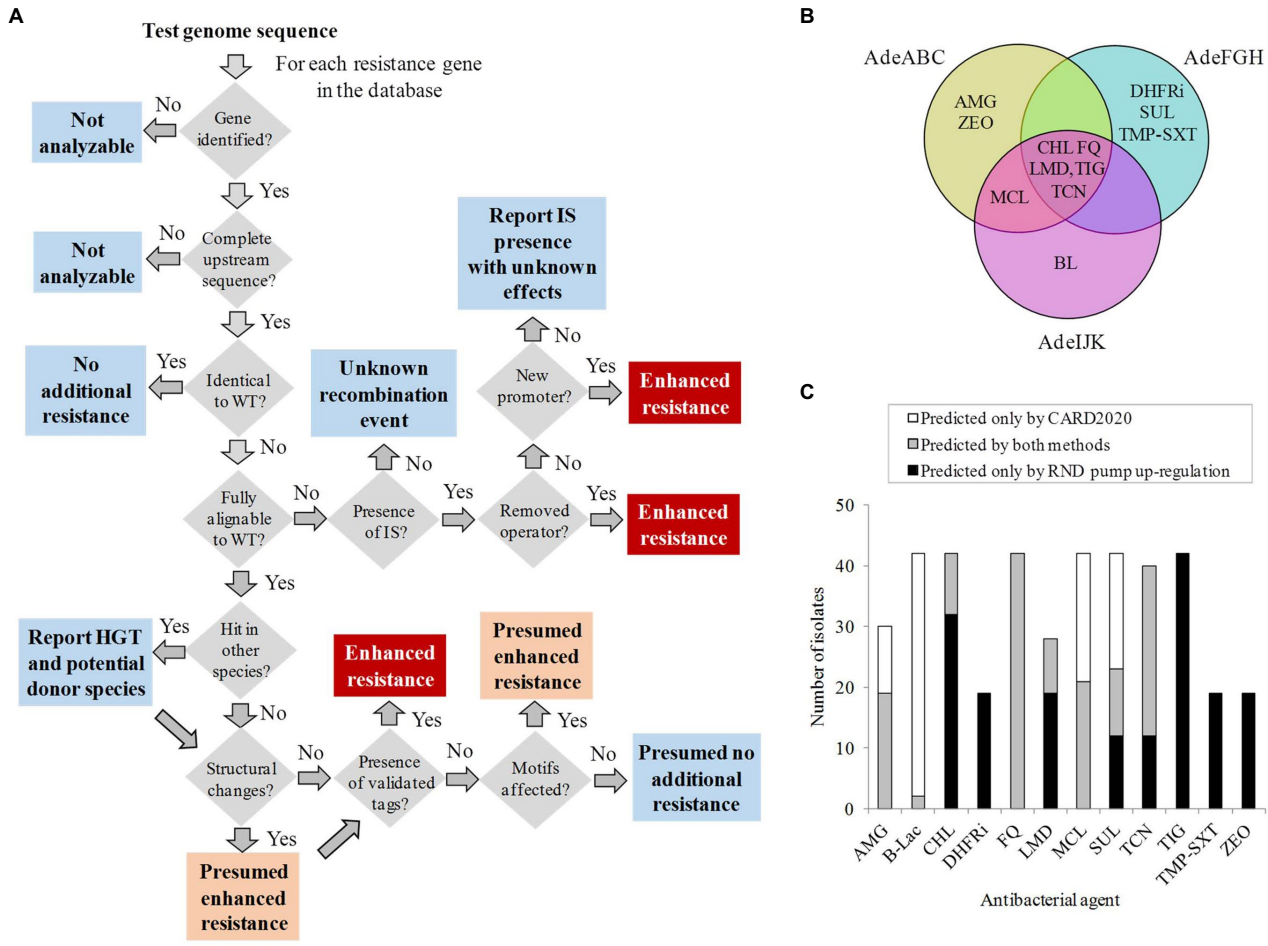
Prediction of the non-coding regulatory resistome. **(A)** Information management flowchart built with decision (grey), warning (light blue), presumed resistance (orange), and verified resistance (red) boxes. **(B)** Venn diagram with the antibacterial class descriptors associated with resistance for each RND pump. AMG, aminoglycosides; BL, beta-lactams; CHL, chloramphenicol; DHFRi, dihydrofolate reductase inhibitors; FQ, fluoroquinolones; LMD, lincosamides; MCL, macrolides; SUL, sulfonamides; TCN, tetracyclines; TIG, tigecycline; TMP-SXT, trimethoprim sulfamethoxazole; and ZEO, zeocin. **(C)** Exclusivity and concordance between CARD2020 and regulatory resistome predictions for 12 antibacterial agents in the 42 isolates at the “Verified” level of our system.

The results described above would find their utility in clinical practice as an automated resistome tool that identifies genetic tags resulting in increased expression of RND pumps in *A. baumannii*. For that, a procedural flowchart that processes all the upstream sequence information in a sequential order compatible with data structure is proposed (Figure 3A). Briefly, if known markers are detected in the upstream RND sequences of a query genome using this protocol, potential upregulation and subsequently reduced response to antimicrobial therapy may be assigned to the isolate. In these cases, upregulation would be suggested at three certainty levels (“Verified,” “Presumed,” and “Probably irrelevant”), according to the genetic tag identified.

The corresponding response to precise antimicrobial therapy of a clinical isolate based on the detected determinants would be carried out using a controlled vocabulary. For that, the efflux-pump upregulation ontology was completed with a list of antibacterial agents expelled by each upregulated RND pump considered here. This information was exhaustively collected from clinical *A. baumannii* isolates and laboratory studies reported in the literature (Supplementary Table S4). The search accounted for 115 pump-to-phenotype causal associations revealed in 16 articles involving 40 antibacterial agents from 12 classes (Figure 3B). These associations between RND pump and expelled antibacterial agents involved at least 4-fold increases in MIC with respect to their susceptible counterpart. RND efflux pumps are linked to several unrelated antibiotics due to their multi-specificity.

A question in point is what degree of novelty can our approach provide with respect to standard, coding sequence-oriented, resistome tools. For that, results concerning the 42 isolates showing verified RND upregulating markers described above were compared to outcomes of CARD2020, a very complete and frequently updated resistome protocol for these isolates (Alcock et al., 2020). Resistance to tigecycline to all 42 isolates was not detected by CARD2020 but associated through RND upstream determinants (Figure 3C). Likewise, new potential resistance to dihydrofolate reductase inhibitors and trimethoprim sulfamethoxazole was assigned to 23 isolates and to zeocin to 19 isolates with verified RND pump upregulation markers. In contrast, several degrees of exclusivity and isolate coverage by validated RND upregulating determinants respect to CARD2020 predictions were obtained for the remaining antibiotic classes, including aminoglycosides and macrolides. Notably, all 42 isolates considered showed co-existing fluoroquinolone efflux prediction and resistance by other mechanisms, suggesting step-wise increases in resistance through several mechanisms for this antibacterial agent class.

Finally, our protocol was applied to 100 phylogenetically unrelated isolates with well-annotated antibiotic resistance and resistome profiles (Galac et al., 2020). Although tigecycline resistance data was not included in this work, we identified that six isolates in the dataset were simultaneously non-susceptible to ciprofloxacin and tetracycline, without a conventional resistome support provided by the curators that justifies these phenotypes. Since ciprofloxacin and tetracycline are substrates of the three principal RND pumps (Yoon et al., 2015), upstream RND sequences of these inconsistent isolates were analyzed in detail. In five of these six cases, isolates carried unusual minor upstream alleles for at least one of the pumps with nucleotide substitutions overlapping or adjacent (<10 positions) to operator and/or promoter elements. All these mutations involved AT::GC changes that modified the DNA bending by 1.5–2.5 degrees per turn on these expression meaning motifs (Supplementary Table S5). These mutations were not experimentally validated but, due to their properties, would be classified in a “Presumed” status according to our scoring system. Notably, four of these mutants harbor the A(-44)G mutation in the −10 promoter box of the *adeIJK* operon. These findings suggest these five isolates are candidate to undergo altered RND efflux, which warrants further experimental investigation.

## Discussion

There is a dramatic lack of bioinformatic strategies that properly approach the regulatory resistome with regard to efflux pumps in multidrug resistant bacteria. This may be explained by the cumbersome regulatory circuitry, involving many heterogeneous aspects that converge into augmented expression of these pumps. Therefore, the identification of clinical isolates carrying efflux-related antibiotic resistance by conventional resistome predictors is prone to either over- or under-detection.

In this study, we provide a catalog of pre-analyzed determinants in upstream regions of the principal RND pumps found in a large genome dataset of *A. baumannii*; in addition to an experimental protocol to screen the influence of the most relevant ones in expression in a timely and cost effective manner; and, finally, a data flowchart that includes a controlled vocabulary between pumps and expelled antibiotics. These three layers may constitute a framework for mature genome-based routine tools. Such tools would predict, with different degrees of certainty, which antibiotic ligands may not achieve clinically-relevant intracytoplasmic levels in a query isolate.

A number of upstream alleles containing a large variety of genetic determinants were found. Different types of genetic events (ISs, indels, and SNPs) were identified, which in some cases may affect expression by directly overlapping key motifs (promoters, operators, and Shine-Dalgarno sequences) and/or nucleic acid structural alterations (DNA curvature and mRNA structure). These markers can be prioritized for experimental validation according to several factors such as the motif/s affected and the prevalence of the marker in the genome dataset. In this regard, the elements with the highest upregulatory confirmed influence for *adeABC* and *adeIJK* operons were ISs. ISs in non-coding upstream sequences have been associated with resistance by overexpression of beta-lactamases/carbapenemases in *A. baumannii* (Corvec et al., 2003; Turton et al., 2006; Adams et al., 2010) and efflux pumps in *Salmonella enterica* (Olliver et al., 2005). ISs are thus versatile genetic elements for *A. baumannii* to modify the efflux response to antibiotics in two different possible ways. Namely, first, by negating repressor loci through intra-gene insertion (Gerson et al., 2018) and, second, by providing potent new promoters to pump genes downstream. However, it should be confirmed whether the same will apply to multidrug bacterial species other than *A. baumannii* or, instead, SNPs in promoter boxes and/or operators are more frequent. This was also the case for *A. baumannii adeFGH*. Upregulation of this operon was proved for mutations in the operator motif and in the Shine-Dalgarno sequence that predictably increase the binding stability to the ribosome. The later result indicates that our protocol may not only be valid for screening enhanced expression markers acting on transcription but also at a translational level. Of note, horizontal transfer of upstream zones from less-pathogenic *Acinetobacter* species showing evidence of different regulation was detected. However, interspecies mosaics produced by recombination potentially affecting pump regulation were extremely rare.

Although some upstream alleles contained several identified determinants, a single-tag classification schema eases analysis, in particular when pertaining to key motifs. However, the combination of several determinants may cooperate to determine the expression phenotype. Thus, a legitimate question is: what should be the subject of study, the single genetic tag or the whole allele, in the upstream sequence resistome? The later could be justified to globally calculate structural properties of nucleic acids that affect expression.

Conventional resistome methods are oriented to the analysis of coding sequences, either gene presence or gene mutations, and in some cases they do not cover pumps. Moreover, genes coding for these RND efflux pumps are present in most *A. baumannii* isolates, irrespective of the efflux-related resistance of the isolate. Therefore, mere gene detection does not suffice for inferring enhanced expression and corresponding resistance, resulting in false positives in isolates that can still be treatable (Grkovic et al., 2001). In our protocol, markers would be labeled as “Verified” if experimentally confirmed as upregulating; “Presumed” if they were not experimentally verified affect key expression motifs; and “Probably irrelevant” for the rest. This escalated certainty assignment of resistance is reminiscent of the BLAST-based “Perfect,” “Strict,” and “Loose” degrees used by formal resistome protocols (Alcock et al., 2020). Likewise, our method resembles the “variant model” (i.e., mutations) rather than the “gene model” (i.e., gene presence) of resistome predictors since it approaches genetic changes that switch the expression modality of core genome genes.

Importantly, and in contrast to other kinds of resistance modes, the upregulating-linked markers concerning RND overexpression may be associated with extended resistance due to the broad range of expelled substrates by RND pumps. However, predictions based on increased RND expression must be interpreted with caution since it may be either (a) as relevant as mutations in primary targets (Lari et al., 2018); (b) be synergic with other mechanisms to quantitatively increase resistance (e.g., from low- to mid- or high-resistance levels; Suh et al., 2010; Schmalstieg et al., 2012); or (c) not or barely contributing to resistance. In particular, our protocol would play a relevant role in the prediction of resistance to tigecycline and of high-level synergistic resistance for fluoroquinolones.

Regarding potential limitations, the relevance of the markers found is interpreted according to current knowledge of expression for the RND pumps analyzed, which may be incomplete. A further technical drawback is that the actual resistance phenotype was not confirmed by introducing the markers directly into the chromosome. Unfortunately, this limitation reflects the scarcity of scalable molecular tools for *A. baumannii*, in particular, for scalable mutation screening in practice. Instead, we have used a state-of-the-art reporter plasmid to detect upregulation since a robust direct correlation between the level of upregulation of the RND genes and the MIC for the antibiotics the pumps expel has been reported (Ruzin et al., 2010). However, our protocol would greatly benefit from ideal high-throughput genome mutagenesis and direct MIC measurement of the resulting strains. Nevertheless, the global schema presented here is dynamic in nature. Thus, future versions of the schema will incorporate additional RND promoters/operators reported in the literature and novel molecular manipulating tools when available for this difficult-to-handle microorganism. Despite the drawbacks, our protocol could be instrumental to check batches of tens of markers associated with irregular enhanced RND expression. This may be a filter step prior to undertake laborious experiments that confirm or rule out antibacterial resistance of the pertaining isolates. We recommend that the plasmid-based screening should be restricted to those markers showing theoretical evidence using the comprehensive catalog of computationally pre-analyzed determinants built here.

In summary, our protocol provides a conceptual predictive upgrade of the resistome tailored to the nature of efflux pump upregulation. The initial *A. baumannii* RND model proposed lays the foundation for knowledge-based identification of efflux pump upregulation. We envisage that it can be broadened to cover more genetic alterations, other prioritized multi-resistant microorganisms and key residue changes in regulators to build a public resource that universally addresses the non-coding regulatory resistome. When applied in combination with regular resistome predictors, this information may help to support genome-guided treatment to prevent ineffective therapy involving fatal consequences. This information may even guide the design of complex synergic therapies that combine canonical antibiotics and anti-efflux drugs (Verma and Tiwari, 2018). In particular, it may be particularly useful for outbreak emerging lineages for which formal resistomes do not match the antibiogram or the therapeutic response.

## Data Availability Statement

The original contributions presented in the study are included in the article/Supplementary Material, further inquiries can be directed to the corresponding author.

## Author Contributions

AM-G conceived the study and performed the computational analyses. AM-G and MM supervised the study, drafted the manuscript, and granted funding. ML-S performed the plasmid constructions and the quantitative analysis of expression. AM-G, MM, and ML-S revised the final version of the manuscript. All authors contributed to the article and approved the submitted version.

## Funding

This research was supported by grants MPY 380/18 and MPY 509/19 from the Instituto de Salud Carlos III (ISCIII). ML-S is the recipient of a Sara Borrell contract by the ISCIII. AM-G is the recipient of a Miguel Servet contract by the ISCIII.

## Conflict of Interest

MM is a founder and shareholder in the biotechnology company Vaxdyn, S.L.

The remaining authors declare that the research was conducted in the absence of any commercial or financial relationships that could be construed as a potential conflict of interest.

## Publisher’s Note

All claims expressed in this article are solely those of the authors and do not necessarily represent those of their affiliated organizations, or those of the publisher, the editors and the reviewers. Any product that may be evaluated in this article, or claim that may be made by its manufacturer, is not guaranteed or endorsed by the publisher.

## Footnotes

^1^ https://pubmlst.org
