# Supplementary material for: Identification of Promoter Region Markers Associated With Altered Expression of Resistance-Nodulation-Division Antibiotic Efflux Pumps in Acinetobacter baumannii
Source: Front Microbiol. 2022 May 19;13:869208. doi: 10.3389/fmicb.2022.869208 (PMC9161033; doi:10.3389/fmicb.2022.869208)
Supplement: Supplementary file 2 [file Data_Sheet_2.PDF]

Table S2. RND upstream alleles involving potential horizontal DNA transfer.

Gene	Index	Number of SNPs respect to WT	Number of isolates	STs[a]	Putative donor species	% Identity donor[b]	% Identity to *A. baumannii*[b]	% ANI respect to putative donor species[c]	% ANI respect to *A. baumannii*[d]
adeA	1	44	4	NF, ST948, ST958	*A. nosocomialis*	100	92	98	92
	2	42	4	NF	*A. nosocomialis*	98	92	92	98
	3	42	1	ST1085	*A. nosocomialis*	99	92	97	92
	4	41	1	ST1343	*A. nosocomialis*	98	92	97	92
	2	40	1	NF	*A. nosocomialis*	98	92	98	92
	6	38	1	NF	*A. nosocomialis*	99	93	98	92
adeF	1	59	1	NF	*A. pittii*	93	90	88	90
	2	54	1	ST1729	*A. pittii*	99	91	97	88
	3	52	1	NF	*A. pittii*	93	91	94	88
	4	43	1	ST1638	*A. pittii*	99	93	97	88
	5	42	1	NF	*A. pittii*	99	93	97	88
	6	22	1	ST1085	*A. nosocomialis*	100	97	97	92
	7	22	1	NF	*A. nosocomialis*	98	92	98	92
	8	21	6	NF, ST948, ST1343	*A. nosocomialis*	100	96	98	92
	9	20	1	ST1085	*A. nosocomialis*	100	97	98	92
adeI	1	29	1	NF	*A. pittii*	97	94	91	87
	2	29	1	ST1638	*A. pittii*	100	94	97	88
	3	28	1	NF	*A. pittii*	100	94	96	88
	4	28	1	ST1729	*A. pittii*	100	94	97	88

[a] STs. MLST sequence types represented by the isolates carrying the upstream allele. NF: not sequence type found.

[b] Identity percentage respect to potential donor species or *A. baumannii* calculated as the average of the top three BLASTn hits of the species sequences stored in the whole nucleotide collection of the NCBI database. When more than one isolate carried the same allele, only a random representative one was analyzed.

[c] % ANI was calculated between allele isolate representative and *A. nosocomialis* 6411 or *A. pittii* PHEA-2 reference strains.

[c] % ANI was calculated between allele isolate representative and *A. baumannii* ATCC17978 strain.
