# Supplementary material for: Identification of Promoter Region Markers Associated With Altered Expression of Resistance-Nodulation-Division Antibiotic Efflux Pumps in Acinetobacter baumannii
Source: Front Microbiol. 2022 May 19;13:869208. doi: 10.3389/fmicb.2022.869208 (PMC9161033; doi:10.3389/fmicb.2022.869208)
Supplement: Supplementary file 6 [file Data_Sheet_1.pdf]

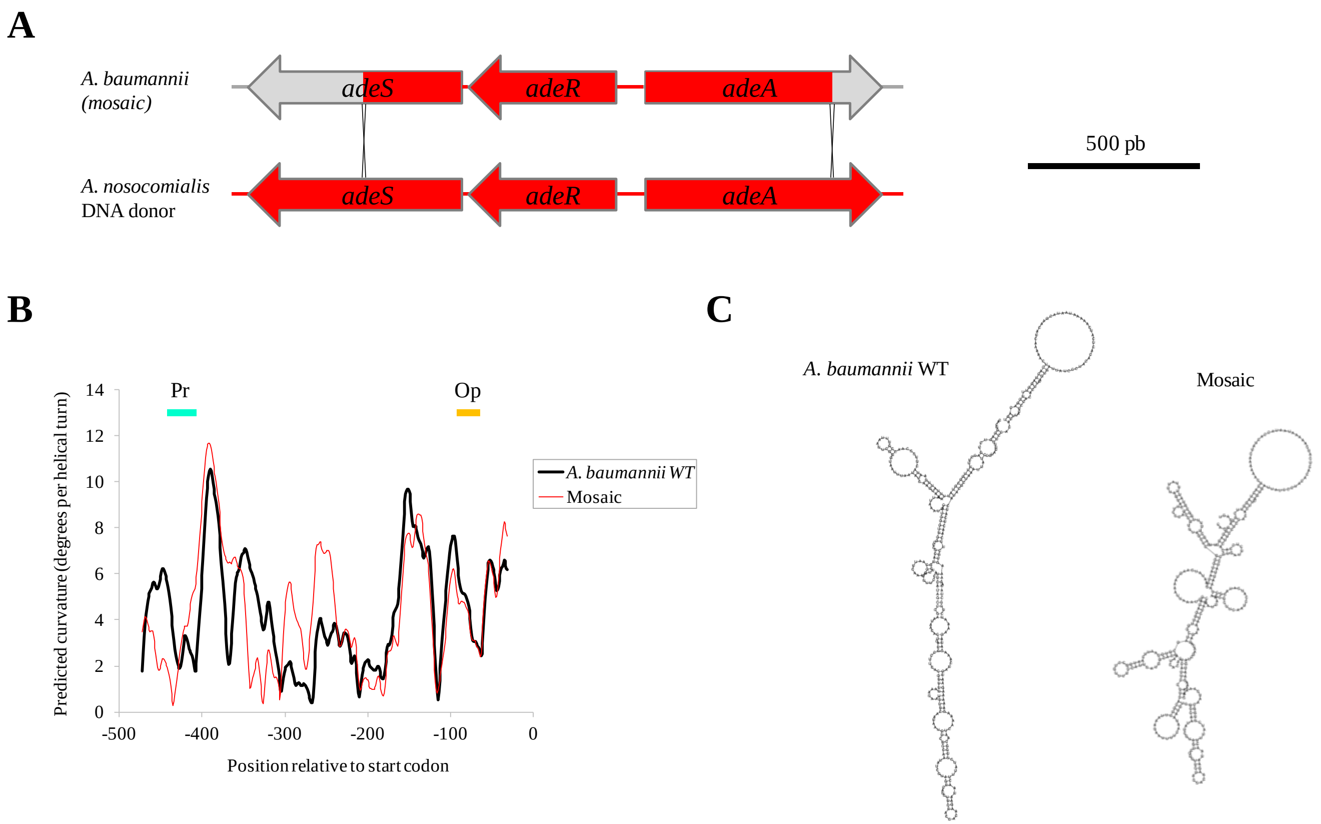

Supplementary Figure S1. Structural properties of the untranslated upstream region of *adeABC* of mosaic (*A. baumannii* / *A. nosocomialis*) Thailand isolates. (A) Genetic architecture schema showing the potential inter-species recombination points in the Thailand mosaic isolates. *A. baumannii* and *A. nosocomialis* sequences are indicated in grey and red, respectively. (B) DNA bending differences between the average of genuine *A. baumannii* alleles (black line) and the mosaic (red line). Promoter (Pr) and operator (Op) sequences are indicated in cyan and orange, respectively. (C) Minimum free energy structures for transcribed untranslated upstream regions (ranging from -403 to -1 positions respect to the start codon) for the upstream *adeA* WT allele (left) and the allele of mosaic isolates (right). Differences between the WT allele and the allele from the mosaic isolates were -60.8 vs -70.2 kcal/mol for the minimum free energy, -69.5 vs -76.0 kcal/mol for the free energy of the thermodynamic ensemble, 91.9 vs 36.3 for the ensemble diversity (average base-pair distance

between all structures in the thermodynamic ensemble) and -42.2 vs -67.3 kcal/mol for the centroid secondary structure (structure with the minimum total base-pair distance to all structures in the thermodynamic ensemble).
